# The Hospital Penny Fund

**Published:** 1904-10-29

**Authors:** 


### "THE HOSPITAL PENNY FUND."

ANOTHER DISCLAIMER.

National Dental Hospital and College,
Great Portland Street, W.

"Dear Sir,—In reply to your letter *re* the Hospital Penny Fund, I am instructed to inform you that the matter will be laid before the next meeting of the committee."

M. P. Collings,
Secretary.

"I may say that the committee has given no authority to use the name of this hospital up to the present time."

October 21st, 1904.

